# Adherence to long-term prophylactic treatment: microeconomic analysis of patients’ behavior and the impact of financial incentives

**DOI:** 10.1186/s13561-019-0222-1

**Published:** 2019-02-13

**Authors:** Klaus Mann, Michael Möcker, Joachim Grosser

**Affiliations:** 1 Department of Psychiatry and Psychotherapy, University Medical Center Mainz, D-55131 Mainz, Germany; 2 Chair of Economic Policy, University of Hagen, D-58084 Hagen, Germany

**Keywords:** Prophylactic treatment, Adherence, Time-inconsistent preferences, Self-control, Financial incentives

## Abstract

The effectiveness of medical therapies depends crucially on patients’ adherence. To gain deeper insight into the behavioral mechanisms underlying adherence, we present a microeconomic model of the decision-making process of an individual who is initially in an asymptomatic clinical state and to whom a prophylactic therapy is offered with the aim of preventing damage to health in the future. The focus of modeling is the optimization of an intertemporal utility function, where time-inconsistent preferences are incorporated by a quasi-hyperbolic discount function. The predictions of the model concur with experience in clinical practice. Moreover, the introduction of time-inconsistency reveals a self-control problem of the individuals where resolutions made before may be given up at a later time. A more pronounced present bias leads to a decrease in adherence and, consequently, the gain in societal welfare resulting from the prophylactic therapy declines. Developing effective strategies to improve adherence is a major challenge in health care. As an example, the impact of financial incentives offered to the patients on adherence and welfare are investigated on the basis of the model. The results are consistent with empirical findings. The approach presented contributes to a better understanding of the complex interaction of the relevant determinants for adherence, particularly regarding the individuals’ self-control problem.

## Introduction

The effectiveness of medical therapies depends crucially on the patient’s adherence, i.e. the extent to which the patient follows the recommendations of a health care provider, such as taking medication, following a diet or executing lifestyle changes. In this regard, many studies have disclosed considerable deficiencies in clinical practice associated with negative consequences for the patients. This particularly holds for the long-term treatment of chronic diseases, with an average estimated adherence rate of 50% [1–4]. Moreover, poor adherence has a considerable economic impact with increased health care costs for the society [5]. Although many different interventions have been described to improve adherence for chronic health conditions, these approaches are usually complex and have not proven to be very effective [6–9].

Because adherence has turned out to be a complex behavior in the context of multiple influencing factors, a profound understanding of this phenomenon is an indispensable requirement for the development of more effective interventions. Adherence can be taken as a decision process regarding the demand for health care goods and, thus, in principle, can be analyzed by applying methods from microeconomics. This approach might yield deeper insight into the behavioral mechanisms underlying adherence [10–17].

In this paper, we present a model of the decision-making process of an individual who is initially in an asymptomatic stage of disease and to whom a therapy is offered with the aim of preventing damage to health in the future. On the one hand, this includes very prevalent chronic pathological conditions such as hypertension, hyperlipidemia, and diabetes mellitus, which may go unnoticed for a long time, but may lead to serious health consequences if no antihypertensive or antidiabetic treatment is carried out in the long run. In addition, this also includes numerous disorders, which are characterized by the absence of symptoms after a temporary episode of illness, but with a high risk of recurrence. Such somatic illnesses are for example the status post-stroke or post-myocardial infarction, which need adequate prophylactic treatment measures after remission to prevent the reoccurrence of ischemic events in the future. The majority of mental disorders also has a high propensity for relapses, e.g. depression and schizophrenia, and, therefore, need adequate maintenance therapy after remission to prevent future episodes of illness.

The extent of the adherence problem and its consequences is illustrated in more detail by the example of hypertension. Although a number of effective antihypertensive drugs are available, numerous studies have shown that a considerable proportion of patients do not take their medication as prescribed, whereby the reported adherence rates vary between studies due to differences in the definition and measurement of adherence and the populations studied. Recent reviews and meta-analyses revealed that 45% [18] and 31% [19], respectively, of hypertensive patients were non-adherent to medication. Another meta-analysis regarding drugs that prevent cardiovascular diseases including several antihypertensive drugs revealed a summary estimate for adherence of 50% in primary prevention and 66% in secondary prevention after a myocardial infarction [20]. There is a large data base demonstrating that lowering blood pressure leads to a reduction of the risk for subsequent adverse outcomes, for meta-analyses see [21–25]. Beside the negative consequences for the affected persons, non-adherence also leads to significant economic burden for the health care system [26–28]. A model analysis on the basis of epidemiological and economic data from five European countries could demonstrate that increasing the adherence rate to antihypertensive medication to 70% would lead to a significant reduction of health care costs [29].

The core element of the decision-making process is the weighting of the various consequences of the pending therapy. The benefits of a prophylactic treatment can only be expected in the future, whereas the costs to be borne by the individual in terms of expenditures of time and money, undesired side effects and emotional distress occur immediately. Against this background, the focus of modeling is the optimization of an intertemporal utility function, which considers the individual’s intertemporal preferences and discounting of future consequences.

While many patients initially consent to the intended therapy, in the long run they often terminate the therapy early contrary to the former agreement. Due to this discrepancy between initial plans and later behavior, non-adherence proves itself as an expression of time-inconsistent behavior. Therefore, to incorporate this deviation from time-consistency, we modify the established discounted utility model traditionally applied for the analysis of intertemporal choice in economics by replacing exponential discounting with a quasi-hyperbolic discount function [30]. Apart from that, individuals are regarded as rationally behaving subjects who aim at maximizing their intertemporal utility using all available information.

The remainder of the paper is structured as follows. First, the individual’s behavior is modeled. If time-inconsistency is allowed in the form of a present bias, then non-adherence appears as an understandable phenomenon from the patient’s viewpoint. Second, the consequences of adherence on the individual and societal welfare are analyzed. Third, to give an example of how worsening of adherence due to time-inconsistency could be counteracted by changing the framework of health care delivery, the impact of financial incentives offered to patients on adherence and individual and societal welfare are investigated. Finally, we discuss the model against both clinical and psychological backgrounds, show the limitations and point out further extensions and applications of the model.

## Modeling of individual behavior

**Fig. 1:**
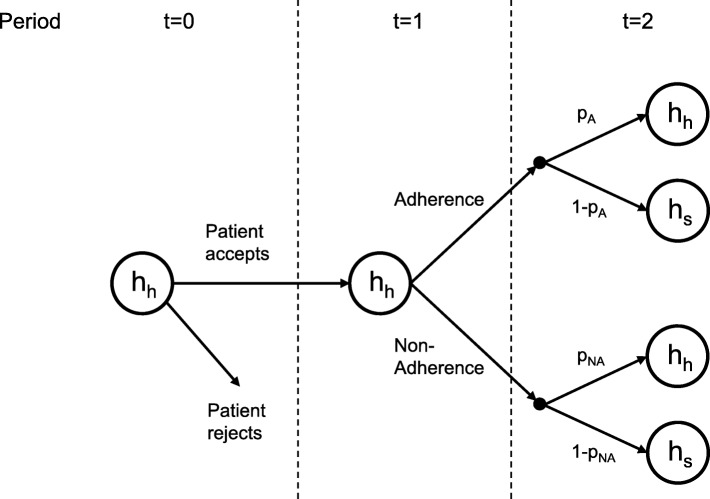
Model of a prophylactic treatment. The model is based on three time periods *t* = 0, 1, 2 with health states *h*_*h*_, *h*_*s*_ and constant transition probabilities *p*_*A*_, *p*_*NA*_ for adherence and non-adherence, respectively. Because the medical concept of adherence implies acceptance of the treatment contract [1], the consequences of rejection of the contract are not shown in detail. (Under the assumptions made, the path after rejection is identical to the path after acceptance followed by non-adherence)

We set up a simple model based on three time periods where only two health states, ‘healthy’ (*h*_*h*_) or ‘sick’ (*h*_*s*_), are considered (Fig. 1). The term ‘healthy’ refers to a clinical condition that is asymptomatic but that requires long-term therapy to prevent the occurrence of symptoms, i.e. transition to the ‘sick’ state. At *t* = 0, the individual is in a healthy state (*h*_*h*_) and enters into a treatment contract. In the following period *t* = 1, the individual is assumed to be still healthy (*h*_*h*_), and he decides either to take the therapy as recommended (adherence) or to refuse it (non-adherence). The health state in period *t* = 2 depends on his behavior in the preceding period. If the individual had undergone the therapy, then the probability to stay further in a healthy state is *p*_*A*_, and the probability to fall ill is 1 − *p*_*A*_. Otherwise, in the case of non-adherence, the probability to remain healthy is reduced to *p*_*NA*_ < *p*_*A*_ and the probability of illness 1 − *p*_*NA*_ is increased.

The decision outcome in period *t* = 1 is particularly determined by the costs to be borne by the individual due to treatment. We assume that treatment is free of charge so that in the case of adherence only non-monetary costs occur, whereas in the case of non-adherence no costs arise (all monetary costs are borne by the society). The non-monetary costs, e.g. in the form of expenditures of time, undesired side-effects and emotional distress, show a large diversity in value among individuals. According to the approach taken by DellaVigna and Malmendier [31] who have analyzed the contract design of a profit-maximizing firm if consumers have time-inconsistent preferences, these costs *c* are modeled as a random variable with distribution *F*(*c*) and density function *f*(*c*), whereby the individual learns his cost type in period *t* = 0 after having accepted the treatment contract.

The instantaneous expected utility *u*_*t*_ experienced by the individual in periods *t* = 1, 2 results from the evaluation of the health state by the individual in the respective periods: *u*_1_ = *u*(*h*_*h*_), ${u}_2^A={p}_A\bullet u\left({h}_h\right)+\left(1-{p}_A\right)\bullet u\left({h}_s\right)$ for adherence and ${u}_2^{NA}={p}_{NA}\bullet u\left({h}_h\right)+\left(1-{p}_{NA}\right)\bullet u\left({h}_s\right)$ for non-adherence, respectively. The intertemporal utility at a given time is calculated as the sum of the discounted instantaneous utilities in succeeding periods.

Various approaches have been developed for formal description of intertemporal preferences [30]. The traditional discounted utility model proposed by Samuelson [32] is characterized by exponential discounting resulting in time-consistent behavior, i.e. the decision outcome does not depend on when the decision is made. However, empirical studies have revealed various deviations of real decision-making behavior from the predictions of this model. In particular, in many cases, hyperbolic discounting can be observed with decreasing discount rates over time [30, 33–36]. As a consequence, regarding decisions to be made in the distant future, the individual behaves patiently pursuing his long-term goals, whereas when the time of making the decision is approaching, an increasing conflict arises between the long-term plans and current temptations. The cause of this time-inconsistency is a relatively high preference for the present with a stronger weighting of immediately occurring consequences compared with consequences in the distant future.

Based on these facts, we incorporate time-inconsistent preferences by a quasi-hyperbolic discounting function. In addition to standard exponential discounting by a factor *δ*, the present period gets the weight 1 and all succeeding periods are weighted by the constant factor 0 < *β* ≤ 1. Accordingly, the intertemporal utility function at a given time *t* is ${U}_t={u}_t+\beta \bullet \sum \limits_{k=1}^{T-1}{\delta}^k\bullet {u}_{t+k}$, where *T* periods are considered [30]. This approach goes back to Phelps and Pollak [37] and has been beneficially applied to various fields of decision making characterized by dynamic inconsistency [31, 38–41].

In the following, the decision-making process in period *t* = 1 is analyzed from two different perspectives ( *t* = 0 and *t* = 1). From the perspective of period *t* = 0, when the individual has entered the treatment contract, the intertemporal utility *U*_0_ for adherence and non-adherence, respectively, is given by

$$ {U}_0^A=-\beta \delta \bullet c+\beta \delta \bullet u\left({h}_h\right)+\beta {\delta}^2\bullet \left[{p}_A\bullet u\left({h}_h\right)+\left(1-{p}_A\right)\bullet u\left({h}_s\right)\right] $$

$$ {U}_0^{NA}=\beta \delta \bullet u\left({h}_h\right)+\beta {\delta}^2\bullet \left[{p}_{NA}\bullet u\left({h}_h\right)+\left(1-{p}_{NA}\right)\bullet u\left({h}_s\right)\right] $$

The individual would decide at *t* = 1 in favor of adherence if the intertemporal utility in the case of adherence turns out to be larger than or equal to the case of non-adherence: ${U}_0^A\ge {U}_0^{NA}$. Thus, the individual would like to be adherent at *t* = 1 if

$$ c\le \delta\ \Delta  p\ \Delta  u $$

where the abbreviations Δu = u(*h*_*h*_) − *u*(*h*_*s*_) and Δ*p* = *p*_*A*_ − *p*_*NA*_ are used. Regarding the whole population of all individuals who have entered into the treatment contract, the probability of adherence from the perspective of *t* = 0 is

$$ F\left(\delta\ \Delta  p\ \Delta  u\right)={\int}_0^{\delta\ \Delta  p\ \Delta  u}f(c)\  dc $$

Beside the distribution of costs *c*, adherence depends on three parameters: adherence is higher with more effective therapy (expressed by *∆p*), larger difference *∆u* between the evaluation of the healthy and sick states by the individual and less exponential discounting of future consequences.

However, the individual’s actual behavior in period *t* = 1 is determined by his evaluation of the consequences at this time. From the perspective of period *t* = 1, when the individual decides whether he undergoes or declines the therapy, the intertemporal utility *U*_1_ for adherence and non-adherence, respectively, is given by

$$ {U}_1^A=-c+u\left({h}_h\right)+\beta \delta \bullet \left[{p}_A\bullet u\left({h}_h\right)+\left(1-{p}_A\right)\bullet u\left({h}_s\right)\right] $$

$$ {U}_1^{NA}=u\left({h}_h\right)+\beta \delta \bullet \left[{p}_{NA}\bullet u\left({h}_h\right)+\left(1-{p}_{NA}\right)\bullet u\left({h}_s\right)\right] $$

From this, as a criterion for adherence we have

$$ c\le \beta \delta\ \Delta  p\ \Delta  u $$

and the probability of adherence is

$$ F\left(\beta \delta\ \Delta  p\ \Delta  u\right)={\int}_0^{\beta \delta\ \Delta  p\ \Delta  u}f(c)\  dc $$

For time-consistent preferences with *β* = 1, the decision outcome in period *t* = 1 is identical to that in the preceding period *t* = 0, i.e. the individual keeps to his earlier resolve. In contrast, in the case of time-inconsistent preferences with *β* < 1, the probability of adherent behavior in period *t* = 1 has decreased compared with the evaluation in period *t* = 0. Under the assumption that *F*(*c*) is a strictly positive monotonic function, we have *F*(*βδ ∆p ∆u*) < *F*(*δ ∆p ∆u*). This is an expression for a self-control problem of the individual. Due to changed preferences, the plan that had originally been considered to be optimal for future behavior is no longer optimal from the perspective of the later decision point of time. That is why the resolution made in the period before is given up at a later time. The less *β* is, the more the actual behavior deviates from the desirable in the long-term optimal behavior, i.e. the more severe is the self-control problem of the individual.

## Welfare analysis

To assess the impact of adherence on the society, we define the joint welfare function *W* = *W*^*I*^ + *W*^*S*^, where *W*^*I*^ is the welfare of a representative individual from the affected population and *W*^*S*^ is the monetary welfare component of the society resulting from treatment costs. Each summand of the welfare function is defined as the expected net utility from the perspective of period *t* = 1, i.e. the benefit after accepting the treatment contract less the remaining benefit in the case that the contract had been rejected (in the case of rejection, the individual’s benefit is ${U}_1^{NA}$, see Fig. 1).

The welfare of an individual is (with expectation operator *E*)

$$ {W}^I=F\left(\beta \delta\ \Delta  p\ \Delta  u\right)\bullet E\left[{U}_1^A-{U}_1^{NA}\right] $$

$$ =F\left(\beta \delta\ \Delta  p\ \Delta  u\right)\bullet \left(-{E}^A\left[c\right]+\beta \delta\ \Delta  p\ \Delta  u\right) $$

$$ =-{\int}_0^{\beta \delta\ \Delta  p\ \Delta  u}c\ f(c)\  dc+F\left(\beta \delta\ \Delta  p\ \Delta  u\right)\bullet \beta \delta\ \Delta  p\ \Delta  u $$

where the expected costs in the case of adherence, *E*^*A*^[*c*], are calculated in the interval *c* ∈ [0, *βδ ∆p ∆u*], yielding ${E}^A\left[c\right]=1/F\left(\beta \delta\ \Delta  p\ \Delta  u\right)\bullet {\int}_0^{\beta \delta\ \Delta  p\ \Delta  u}c\ f(c)\  dc$.

The first term in the equation above is negative and represents the costs to be borne by the individual. The second positive term constitutes the individual’s benefit from the therapy to be expected in the future. For further statements, the distribution of *c* must be specified. Supposing a uniform distribution of costs in the interval *c* ∈ [0, *c*_*max*_] with *f*(*c*) = 1/*c*_*max*_ and *F*(*c*) = *c*/*c*_*max*_, the individual’s welfare can be written as

$$ {W}^I=-\frac{1}{c_{max}}{\int}_0^{\mathit{\min}\ \left(\beta \delta\ \Delta  p\ \Delta  u,{c}_{max}\right)}c\  dc+\frac{\ \mathit{\min}\ \left(\beta \delta\ \Delta  p\ \Delta  u,{c}_{max}\right)}{c_{max}}\bullet \beta \delta\ \Delta  p\ \Delta  u $$

$$ =\left\{\begin{array}{l}\frac{{\left(\beta \delta\ \Delta  p\ \Delta  u\right)}^2}{2\ {c}_{max}}\kern4em when\kern1em \beta \delta\ \Delta  p\ \Delta  u<{c}_{max}\\ {}-\frac{c_{max}}{2} + \beta \delta\ \Delta  p\ \Delta  u\kern1.5em when\kern1em \beta \delta\ \Delta  p\ \Delta  u\ge {c}_{max}\end{array}\right. $$

Of particular importance is the individual’s self-control, which is represented by the parameter *β* in the model. For *δ ∆p ∆u* < *c*_*max*_, i.e. adherence from the perspective of period *t* = 0 is not complete in the population, with falling *β*, the expected gain in welfare shows a quadratic decrease from a maximum of (*δ ∆p ∆u*)^2^/2 *c*_*max*_ for *β* = 1 to 0 for *β* = 0. The smaller *β* is, the less is adherence and the more the welfare gain deviates from the optimum in the case of full self-control.

The monetary welfare of the society is determined by the expenses due to the prophylactic therapy in period *t* = 1, *C*_*A*_ and *C*_*NA*_ in the case of adherence and non-adherence, respectively, where *C*_*NA*_ < *C*_*A*_ is assumed, as well as by the follow-up costs *C*_*F*_ incurred by individuals falling ill in period *t* = 2, containing both medical costs and indirect costs due to productivity loss. Considering the society’s costs in the case of contract rejection (1 − *p*_*NA*_) ∙ *δ C*_*F*_, the monetary societal welfare is

$$ {W}^S=-F\left(\beta \delta\ \Delta  p\ \Delta  u\right)\bullet \left[{C}_A+\left(1-{p}_A\right)\bullet \delta\ {C}_F\right]-\left(1-F\left(\beta \delta\ \Delta  p\ \Delta  u\right)\right)\bullet \left[{C}_{NA}+\left(1-{p}_{NA}\right)\bullet \delta\ {C}_F\right]+\left(1-{p}_{NA}\right)\bullet \delta\ {C}_F $$

$$ =-F\left(\beta \delta\ \Delta  p\ \Delta  u\right)\bullet {C}_A-\left(1-F\left(\beta \delta\ \Delta  p\ \Delta  u\right)\right)\bullet {C}_{NA}+F\left(\beta \delta\ \Delta  p\ \Delta  u\right)\bullet \Delta  p\bullet \delta\ {C}_F $$

where the society’s discounting factor is assumed to be the same as for the individual.

The first two summands reflect the prophylactic treatment costs for adherent and non-adherent individuals, respectively, in period *t* = 1, and the third term represents the savings on follow-up costs incurred by individuals not falling ill in period *t* = 2 due to prophylactic treatment. There are opposite effects with respect to adherence. With falling *β*, the treatment costs in period *t* = 1 decrease (assuming *C*_*A*_ > *C*_*NA*_), whereas the burden on society due to cases of illness in period *t* = 2 increases. The net effect is determined by the relationship between the expenses in period *t* = 1 and the follow-up costs in period *t* = 2. Under the assumption that the follow-up costs in cases of illness in period *t* = 2 are high compared with the treatment costs in period *t* = 1, *W*^*S*^ is positive because cases of illness can be prevented by prophylactic therapy and a decrease of *β* leads to a decline of *W*^*S*^. This can be seen from the partial derivative *∂W*^*S*^/*∂β* =  − *f*(*βδ ∆p ∆u*) ∙ *δ ∆p ∆u* ∙ (*C*_*A*_ − *C*_*NA*_ − *∆p* ∙ *δ C*_*F*_), which is positive if the follow-up costs in period *t* = 2 are sufficiently high compared to the treatment costs in period *t* = 1 and the density function *f*(*βδ ∆p ∆u*) is greater than 0 (referring to the above analysis, assuming a uniform distribution of costs, this is the case if adherence is not complete).

Under the assumptions made, both the individual’s welfare and monetary societal welfare are positive. However, the gain in welfare is restricted by individuals’ self-control problem. The less *β* is, the less is the increase of welfare caused by the chance of prophylactic treatment. Against this background, the search for measures to reduce the self-control problem and, thus, to improve adherence is not only worthwhile from the individuals’ viewpoint but also from the perspective of society.

## Impact of financial incentives on adherence and welfare

Starting from the model presented above, we now assume that adherent patients are rewarded with a monetary bonus in the amount of *b* > 0. However, as the individual’s behavior can be observed only to a limited extent, an improper claim for a bonus put in by non-adherent individuals has to be considered. If the probability that a bonus abuse will be detected is *p*_*D*_ and under the assumption that moral aspects are no object, then the intertemporal utility functions from the perspective of periods *t* = 0 and *t* = 1, respectively, each for adherence and non-adherence, will take the form

$$ {U}_0^A=-\beta \delta \bullet \left(c-b\right)+\beta \delta \bullet u\left({h}_h\right)+\beta {\delta}^2\bullet \left[{p}_A\bullet u\left({h}_h\right)+\left(1-{p}_A\right)\bullet u\left({h}_s\right)\right] $$

$$ {U}_0^{NA}=\beta \delta \bullet \left(1-{p}_D\right)\bullet b+\beta \delta \bullet u\left({h}_h\right)+\beta {\delta}^2\bullet \left[{p}_{NA}\bullet u\left({h}_h\right)+\left(1-{p}_{NA}\right)\bullet u\left({h}_s\right)\right] $$

$$ {U}_1^A=-\left(c-b\right)+u\left({h}_h\right)+\beta \delta \bullet \left[{p}_A\bullet u\left({h}_h\right)+\left(1-{p}_A\right)\bullet u\left({h}_s\right)\right] $$

$$ {U}_1^{NA}=\left(1-{p}_D\right)\bullet b+u\left({h}_h\right)+\beta \delta \bullet \left[{p}_{NA}\bullet u\left({h}_h\right)+\left(1-{p}_{NA}\right)\bullet u\left({h}_s\right)\right] $$

According to the former argument, the criteria for adherence from the perspective of period *t* = 0 and *t* = 1, respectively, are

$$ c\le \delta\ \Delta  p\ \Delta  u+{p}_D\ b $$

$$ c\le \beta \delta\ \Delta  p\ \Delta  u+{p}_D\ b $$

and the corresponding probabilities of adherence are

$$ F\left(\delta\ \Delta  p\ \Delta  u+{p}_D\ b\right)={\int}_0^{\delta\ \Delta  p\ \Delta  u+{p}_Db}f(c)\  dc $$

$$ F\left(\beta \delta\ \Delta  p\ \Delta  u+{p}_D\ b\right)={\int}_0^{\beta \delta\ \Delta  p\ \Delta  u+{p}_Db}f(c)\  dc $$

Due to *dF*/*db* = *f*(*βδ ∆p ∆u* + *p*_*D*_ *b*) ∙ *p*_*D*_ > 0, the prospect of a bonus leads to an increase of adherence. The higher the amount of the bonus *b* and the higher the probability of detection *p*_*D*_, the higher is the probability of adherent behavior. In the case of complete observability of the individuals’ behavior (*p*_*D*_ = 1), the entire amount of the bonus comes to fruition, and adherence will be maximized. For *p*_*D*_ = 0, the bonus will be equally distributed among all individuals, and adherence will remain unchanged.

Instead of rewarding adherent behavior, non-adherence could alternatively be sanctioned. This can be formalized analogously by additional costs in period *t* = 1 in the event of non-adherence. The result is identical with an increase of adherence depending on the amount of sanction and the probability that non-adherence will be detected.

For welfare analysis, it must be considered that if the treatment contract is rejected, no bonus will be paid out, whereas non-adherent individuals will be in receipt of the bonus at least partially, accompanied by an increase of their utility in the amount of (1 − *p*_*D*_) ∙ *b*. After accepting the treatment contract, the welfare of an individual is

$$ {W}^I=F\left(\beta \delta\ \Delta  p\ \Delta  u+{p}_D\ b\right)\bullet E\left[{U}_1^A-{U}_1^{NA}\right]+\left(1-{p}_D\right)\bullet b $$

$$ =F\left(\beta \delta\ \Delta  p\ \Delta  u+{p}_D\ b\right)\bullet \left(-{E}^A\left[c\right]+\beta \delta\ \Delta  p\ \Delta  u+{p}_D\ b\right)+\left(1-{p}_D\right)\bullet b $$

$$ =-{\int}_0^{\beta \delta\ \Delta  p\ \Delta  u+{p}_Db}c\ f(c)\  dc+F\left(\beta \delta\ \Delta  p\ \Delta  u+{p}_D\ b\right)\bullet \left(\beta \delta\ \Delta  p\ \Delta  u+{p}_D\ b\right)+\left(1-{p}_D\right)\bullet b $$

Supposing a uniform distribution of costs in the interval *c* ∈ [0, *c*_*max*_], according to the former calculation, the individual’s welfare can be represented by

$$ {W}^I=\left\{\ \begin{array}{l}\frac{{\left(\beta \delta\ \Delta  p\ \Delta  u+{p}_D\ b\right)}^2}{2\ {c}_{max}}+\left(1-{p}_D\right)\bullet b\  when\kern1em \beta \delta\ \Delta  p\ \Delta  u+{p}_D\ b<{c}_{max}\\ {}-\frac{c_{max}}{2} + \beta \delta\ \Delta  p\ \Delta  u+b\kern1.2em when\kern1em \beta \delta\ \Delta  p\ \Delta  u+{p}_D\ b\ge {c}_{max}\end{array}\right. $$

Financial incentives lead to an increase of the individual’s welfare (*∂W*^*I*^/*∂b* > 0), resulting directly from paying out of the bonus and indirectly from a heightened adherence.

The monetary welfare of society comprises the treatment costs in period *t* = 1 and the follow-up costs incurred by individuals falling ill in period *t* = 2, according to the previous modeling. In addition, society makes a payment in terms of the bonus. This is associated with further costs, which increase with a higher targeted probability of detection *p*_*D*_. Under the assumption that these additional costs come to a percentage *λ* of the bonus *b*, we have

$$ {W}^S=-{C}_{NA}-F\left(\beta \delta\ \Delta  p\ \Delta  u+{p}_D\ b\right)\bullet \left({C}_A-{C}_{NA}-\Delta  p\bullet \delta\ {C}_F\right) $$

$$ -\left[F\left(\beta \delta\ \Delta  p\ \Delta  u+{p}_D\ b\right)+\left(1-F\left(\beta \delta\ \Delta  p\ \Delta  u+{p}_D\ b\right)\right)\bullet \left(1-{p}_D\right)\right]\bullet \left(1+\lambda \right)\bullet b $$

Due to higher adherence, the bonus leads to an increase of the net effect of treatment costs and follow-up costs. If the follow-up costs are sufficiently high, then this net effect is positive. However, due to paying out of the bonus and due to the additional costs involved, society’s welfare diminishes.

Total welfare *W* = *W*^*I*^ + *W*^*S*^ is given by

$$ W=-{\int}_0^{\beta \delta\ \Delta  p\ \Delta  u+{p}_Db}c\ f(c)\  dc+F\left(\beta \delta\ \Delta  p\ \Delta  u+{p}_D\ b\right)\bullet \beta \delta\ \Delta  p\ \Delta  u $$

$$ -{C}_{NA}-F\left(\beta \delta\ \Delta  p\ \Delta  u+{p}_D\ b\right)\bullet \left({C}_A-{C}_{NA}-\Delta  p\bullet \delta\ {C}_F\right) $$

$$ -\left[F\left(\beta \delta\ \Delta  p\ \Delta  u+{p}_D\ b\right)+\left(1-F\left(\beta \delta\ \Delta  p\ \Delta  u+{p}_D\ b\right)\right)\bullet \left(1-{p}_D\right)\right]\bullet \lambda b $$

The bonus paid to the individuals is a redistribution on a scale of *F*(*βδ ∆p ∆u* + *p*_*D*_ *b*) ∙ *b* + (1 − *F*(*βδ ∆p ∆u* + *p*_*D*_ *b*)) ∙ (1 − *p*_*D*_) ∙ *b* within the system, which does not affect total welfare. However, all other components change because the probability of adherence increases and additional costs arise due to paying out the bonus. Supposing a uniform distribution of costs in the interval *c* ∈ [0, *c*_*max*_] and restricting to the condition that adherence is not complete from the perspective of period *t* = 0 in the population (*βδ ∆p ∆u* + *p*_*D*_ *b* < *c*_*max*_), as well as under the simplifying assumption of complete observability of the individuals’ behavior (*p*_*D*_ = 1), it follows

$$ W=\frac{1}{2\ {c}_{max}}\ \left[{\left(\beta \delta\ \Delta  p\ \Delta  u\right)}^2-2\ \beta \delta\ \Delta  p\ \Delta  u\bullet \left({C}_A-{C}_{NA}-\Delta  p\ \delta\ {C}_F\right)-2\ \left({C}_A-{C}_{NA}-\Delta  p\ \delta\ {C}_F+\beta \delta\ \Delta  p\ \Delta  u\ \lambda \right)\bullet b-\left(1+2\ \lambda \right)\bullet {b}^2\right]-{C}_{NA} $$

Assuming that the follow-up costs *C*_*F*_ are high compared with the prophylactic treatment costs, and provided that the additional costs caused by managing the bonus payment are not too high (*λ* < (*δ ∆p C*_*F*_ − (*C*_*A*_ − *C*_*NA*_))/(*βδ ∆p ∆u*)), total welfare increases due to the bonus, according to a concave parabolic course. Then, the optimal amount of the bonus leading to maximum welfare results from the first-order condition *∂W*/*∂b* = 0:

$$ {b}^{\ast }=\frac{\delta\ \Delta  p\ {C}_F-\left({C}_A-{C}_{NA}\right)-\beta \delta\ \Delta  p\ \Delta  u\ \lambda }{1+2\ \lambda } $$

In several health systems, patients have to make out-of-pocket co-payments when they demand health care services; thus, the bonus could be realized in the form of a reduction of this co-payment. By slight modification of the model presented above, the consequences of co-payment can be analyzed analogously. In addition to the costs *c*, the co-payment has to be considered in period *t* = 1 for all adherent and partly also for non-adherent patients, which is equivalent to a negative bonus. Implementation of co-payment leads to a decrease of adherence and individual welfare. Regarding societal welfare, the opposite effects become obvious. The co-payment, less the cost of administration, benefits the society, whereas the gain in societal welfare as a result from treatment is reduced because of the worse outcome due to diminished adherence.

## Discussion

We have presented a microeconomic model of the adherence behavior of an individual, who is in an asymptomatic stage of disease and to whom a therapy is offered to prevent damage to health in the future. The model predicts that adherence is higher when impairments due to the therapy are less, the treatment in respect of preventing illness in the future is more effective and the resulting benefit when the individual remains healthy compared to illness is greater. These results concur with experience in clinical practice.

Analyzing decision behavior from different temporal perspectives, the individual’s self-control problem could be identified as a further decisive determinant of adherence, which can particularly serve as an explanation for early discontinuation of the therapy. The present bias, represented by the parameter *β* < 1, implicates time-inconsistent preferences so that the actual decision comes into conflict with the plans made formerly. The smaller *β* is, the larger is the deviation of the actual behavior from the desired long-term optimal behavior, i.e. the more adherence is reduced in the further course of treatment with regard to the original intent. Thus, this approach represents an extension of the application of hyperbolic discounting to the formal description of self-control problems, as has already been done for other intertemporal decision-making situations, which are initially associated with costs and only later promise a benefit [31, 38–41].

Accordingly, other intertemporal choices can also been described, where an immediate benefit is to be expected and costs only occur after a delay. A typical example from medicine is addiction. Based on empirical findings, both the sustained consumption of a substance despite the associated future negative consequences and the loss of control in the form of a relapse despite a previously formulated abstinence goal can be adequately described formally by a hyperbolic discount function [42]. Moreover, further studies, reviewed in [43], have shown increased discount rates in addicts compared to healthy controls and a correlation between the strength of discounting and the severity of addiction. Carrillo [44] has used a quasi-hyperbolic discount function for a theoretical analysis of the decision-making in addiction: while moderate consumption has proven to be the best long-term strategy, excessive consumption occurs in the future when there is a strong propensity to consume. Moreover, he could demonstrate that, if the optimal consumption behavior is not realizable due to a lack of commitment about future behavior, abstinence is the second best strategy offering protection against excessive consumption, provided that discounting is sufficiently strong.

It should be mentioned here that hyperbolic discount functions provide a formal description of time-inconsistent behavior in intertemporal choices, which does not raise the claim to explain the real underlying psychological mechanisms. Rather, on the psychological level, non-adherence appears as a complex phenomenon, probably resulting as a joint product of multiple distinct mechanisms. Beside the concept of time discounting, transient visceral influences as well as personality traits, such as impulsivity or restrictions to exercise self-control, may be relevant psychological factors, e.g., whereby the extent of their contribution varies depending on the individual and the context of decision-making. However, based on empirical data, time discounting has proved to be an important contributing factor in various fields of intertemporal choice [34, 43, 45–52].

An important approach to explain time-inconsistent preferences on the psychological level is provided by the dual process theories, which make a connection to cerebral functions. According to Brocas and Carillo [53], intertemporal decision processes result from a complex interaction of an impulsive system, which is responsible for the judgement of information with regard of immediate consequences, and a reflective system, which makes judgements from a longer-term perspective. These systems are based on different mechanisms of information processing [54, 55]; meanwhile, there are also empirical findings connecting these psychological theories to neurobiological processes in the brain [56–61].

Against the background of these psychological aspects, hyperbolic discounting represents a rather simple model, particularly in the form of a quasi-hyperbolic discount function, which is often used due to its easier analytical tractability. In the literature, several extensions of the hyperbolic discount model have been reported by adding additional arguments to the instantaneous utility function. Regarding addictive behavior, e.g. the concept of habit formation has been proposed, where the utility from current consumption is affected by the extent of past consumption [30]. Another interesting approach has been proposed by Loewenstein [30, 62, 63], which incorporates utility from anticipation. This model assumes that instantaneous utility is determined not only by current consumption, but also depends on anticipating future consumption. This might be a relevant aspect also in the context of treatment adherence due to a greater consideration of the benefits of prevention. Finally, multiple-self models are also to be mentioned which view intertemporal choices as the outcome of a conflict between myopic selves and more farsighted ones, who alternatively take control of behavior [30].

Emanating from the model, several strategies can be derived to promote adherence, particularly to restrict the detrimental consequences of the self-control problem, both on the patient-level and the health care system level. Regarding the physician-patient relationship, optimization of the therapy in the case of insufficient effectiveness or relevant side effects is of particular significance. Because adherence is determined by the patient’s subjective perception of the treatment effects, information delivered by the providers plays a decisive role in terms of both the content and mode of transmission. In this context, the model shows also the area of conflict that the physician is exposed to when informing the patient in clinical routine. On the one hand, the physician is obliged to inform the patient about all relevant side effects and risks; on the other hand, comprehensive information about these unfavorable aspects may release fears and, thereby, reduce adherence. There is also the complicating fact that some patients wish to abstain from additional information in order to not jeopardize their adherence. Such behaviors, which at first glance appear unreasonable, can turn out to be effective coping strategies from the patient’s view. Here, interesting extensions of the model may present themselves for a better understanding of those phenomena [64, 65].

Another approach to improve adherence is to raise the patient’s awareness of his self-control problem. A sophisticated individual can try to bind his actual behavior to his earlier intentions by using commitment strategies to better achieve his optimal goals for the long term. Starting from a paper by Strotz [66], in the previous decades, researchers have pointed to the importance of such commitment strategies [38, 67, 68]. Regarding this aspect, the model may also serve as a starting point to analyze the conditions under which an individual will be prepared to make a commitment concerning treatment that will restrict his freedom of action in the future.

In addition to measures on the individual level, interventions on the health care system level may also contribute to improve adherence. As an example, we have analyzed the consequences of monetary incentives for adherent behavior. Adherence and individual welfare grow with the size of the reward, which concurs with empirical findings [69–72]. Regarding the impact on society, opposite effects occur: societal welfare decreases due to the costs of the preventive measures, whereas the lower incidence of illness resulting in lower costs in the future leads to a gain in welfare. The higher the costs arising from the occurrence of symptomatic illness in the future and the more effective the prophylactic therapy, the larger is the rise in societal welfare. The appropriate amount of the bonus can be determined by maximizing the joint welfare of the individual and the society. However, the prevention of improper use of the bonus requires an effective monitoring system. Examples successfully applied in practice are therapeutic drug monitoring with regularly taking of blood samples to measure the concentration of medication, the implementation of outpatient drug services with daily short-term visits at home to administer medication, and the intramuscular administration of depot-antipsychotics in schizophrenic patients. Similar to a bonus system, the effects of patients’ co-payments for health care utilization can be analyzed. Higher cost sharing is associated with a lower likelihood of adherence, which is also consistent with empirical studies [73–75].

Several limitations have to be considered. The presented model is essentially based on the fundamental assumptions of neoclassical theory that supposes individuals act rationally to maximize their intertemporal benefit. As the only deviation from traditional theory, time-inconsistent preferences have been introduced. However, Simon [76] had already indicated the restrictions of human cognitive capabilities and limited information. In the following decades, behavioral economics research could identify numerous further decision anomalies leading to deviations from traditional theory. Thus, for example, the presentation of the prospect of an incentive may have different effects depending on how the context is designed [77–79]. Besides the fact that intuitive cognitive mechanisms and heuristics are also important components of decision processes [80, 81], emotional aspects in the context of decision making have gained particular attention [82–85]. According to the risk-as-feeling model developed by Loewenstein et al. [86], emotions play a decisive role by both influencing cognitive judgement processes and directing the individual’s behavior. It becomes increasingly accepted now that decision outcomes result from a synergistic interaction of various cognitive and emotional processes. Particularly in the context of medical decisions, the relevance of these aspects is obvious. Regarding adherence, confidence and empathy in the relationship between patient and physician, and the support provided by the social environment play an important role. In many cases, cognitive restrictions also affect medical decision making, particularly in older people or in patients suffering from mental disorders. These influencing factors must be taken into account both in individual decision-making situations and, in particular, in policy recommendations with regard to the enhancement of adherence.

## Conclusions

The predictions of the model are consistent with clinical experience and empirical findings, pointing out that the model includes the relevant determinants for adherence and reflects their complex interaction. Thus, modeling can contribute to a better understanding of this phenomenon. This holds particularly for the self-control problem with a decrease of adherence in the course of treatment, which becomes comprehensible as a consequence of time-inconsistent preferences of otherwise rationally behaving individuals. These insights on the part of health care providers may contribute to a better understanding of patients’ superficially irrational and non-compliant behavior. Regarding measures to improve adherence, modeling allows to assess the consequences of intended interventions in a theoretic way in the run-up to empirical studies or to a broad implementation in clinical routine. However, with regard to policy recommendations for clinical practice, one must be aware of the complexity of the phenomenon of adherence with several influencing factors which are not covered by the neoclassical framework underlying the model. Finally, the approach presented may serve as a starting point for extensions, including explicit modeling of the cognitive and emotional aspects of decision making.

## Abbreviations

*h*_*h*_ and *h*_*s*_: Health states ‘healthy’ and ‘sick’.
A: Adherence
E: Expectation operator
NA: Non-adherence
